# Structural Prediction of the Dimeric Form of the Mammalian Translocator Membrane Protein TSPO: A Key Target for Brain Diagnostics

**DOI:** 10.3390/ijms19092588

**Published:** 2018-08-31

**Authors:** Juan Zeng, Riccardo Guareschi, Mangesh Damre, Ruyin Cao, Achim Kless, Bernd Neumaier, Andreas Bauer, Alejandro Giorgetti, Paolo Carloni, Giulia Rossetti

**Affiliations:** 1 Institute for Advanced Simulations (IAS)-5/Institute for Neuroscience and Medicine (INM)-9, Forschungszentrum Jülich, 52428 Jülich, Germany; j.zeng@fz-juelich.de (J.Z.); r.guareschi@fz-juelich.de (R.G.); caobb0214@gmail.com (R.C.); p.carloni@fz-juelich.de (P.C.); 2 Laboratory of Computational Chemistry and Drug Design, Laboratory of Chemical Genomics, Peking University Shenzhen Graduate School, 518055 Shenzhen, China; 3 Department of Biotechnology, University of Verona, Strada Le Grazie 15, 37134 Verona, Italy; mangeshdamre007@gmail.com; 4 Neurobiology, International School for Advanced Studies (SISSA), 34136 Trieste, Italy; 5 Grünenthal Innovation, Translational Science & Intelligence, Grünenthal GmbH, 52078 Aachen, Germany; Achim.Kless@grunenthal.com; 6 Institute for Neuroscience and Medicine (INM)-5, Forschungszentrum Jülich, 52428 Jülich, Germany; b.neumaier@fz-juelich.de; 7 Institute for Neuroscience and Medicine (INM)-2, Forschungszentrum Jülich, 52428 Jülich, Germany; an.bauer@fz-juelich.de; 8 RWTH Aachen University, Department of Physics, 52078 Aachen, Germany; 9 Jülich Supercomputing Center (JSC), Forschungszentrum Jülich, 52428 Jülich, Germany; 10 University Hospital Aachen, RWTH Aachen University, 52078 Aachen, Germany

**Keywords:** TSPO, radioligands, PET, oligomerization, brain inflammation, homology modeling, molecular dynamics, docking

## Abstract

Positron emission tomography (PET) radioligands targeting the human translocator membrane protein (TSPO) are broadly used for the investigations of neuroinflammatory conditions associated with neurological disorders. Structural information on the mammalian protein homodimers—the suggested functional state of the protein—is limited to a solid-state nuclear magnetic resonance (NMR) study and to a model based on the previously-deposited solution NMR structure of the monomeric mouse protein. Computational studies performed here suggest that the NMR-solved structure in the presence of detergents is not prone to dimer formation and is furthermore unstable in its native membrane environment. We, therefore, propose a new model of the functionally-relevant dimeric form of the mouse protein, based on a prokaryotic homologue. The model, fully consistent with solid-state NMR data, is very different from the previous predictions. Hence, it provides, for the first time, structural insights into this pharmaceutically-important target which are fully consistent with experimental data.

## 1. Introduction

The human translocator membrane protein (TSPO) [1,2,3] is a key biomarker for the diagnostics of inflammatory conditions in the brain [4]. Expression of human TSPO in the outer membrane of the mitochondria is indeed strongly up-regulated in areas of brain injury and in neuroinflammatory conditions, including those associated with Alzheimer’s and Parkinson’s disease [5]. Increased expression levels of human TSPO can be monitored by positron emission tomography (PET). PET uses radiolabeled human TSPO ligands to sensitively recognize lesions and active disease processes of the brain [6,7,8]. In addition, selective human TSPO ligands are expected to be therapeutic agents with a wide spectrum of action against psychiatric disorders and limited side effects [9,10]. Several specific diagnostic ligands show potent activity (K_i_: 0.18–11 nM) for both rat and human TSPOs [11]. More recently, other studies have investigated the use of low-affinity TSPO binders to treat traumatic brain injury and brain inflammation [12,13,14,15]. These works have pointed out that also low-affinity binders of TSPO can be effectively employed to control apoptosis, to reduce neuronal degeneration and promote neurosteroidogenesis.

**Figure 1:**
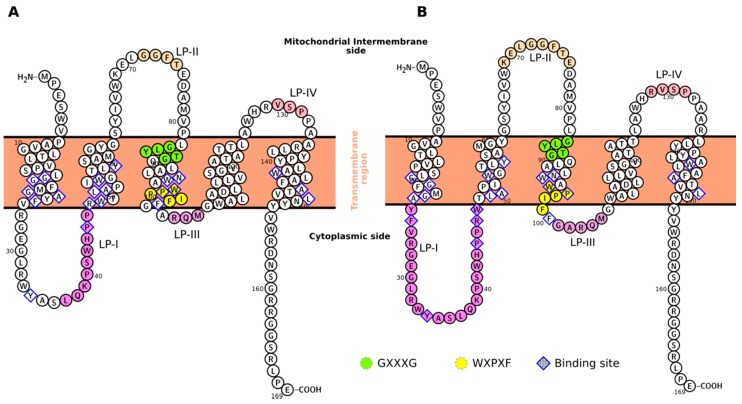
Topology of mTSPO for the models here considered. Specific regions and motifs discussed in the text are highlighted for mTSPO_NMR_monomer (**A**) and mTSPO_Rs_monomer (**B**). The topology is generated using the Protter web application [19].

However, the mode of action of these ligands is still unclear due to the controversial structural information on TSPO. The protein topology consists of five transmembrane helices (TM-I to TM-V) connected by two loops (LP-I and LP-III) at the cytosol interface and two loops (LP-II and LP-IV) placed in the intermembrane space (Figure 1). Mammalian TSPOs can assemble in multimeric complexes (up to six units) in vivo [16,17], but the dimeric form is likely to be already functional [2,18]. Unfortunately, no oligomeric mammalian experimental structure is available.

Current information on the structural determinants and the subunit-subunit assembly of mammalian TSPOs is derived from solid-state NMR investigations on the mouse protein (mTSPO) [20]. This is expected to be similar to the human TSPO, because of the high mutual sequence identity (81%, as obtained from the analysis of the sequence identity performed with BLAST [21,22]). mTSPO’s monomer-monomer interface involves residues from the TM-III helix [20] (Figure 1 and Table 1). These include: (i) the G83XXXG87 (X = any residue) motif present across different protein classes’ interfaces, [23,24,25,26,27]; and (ii) the W95XPXF98 motif [28], occurring across eukaryotic and prokaryotic TSPOs. In the same study, the authors also presented a prediction of the functionally relevant dimer structure, based on a previously published solution NMR structure of monomeric mTSPO [29] in complex with its high affinity ligand 1-(2-chlorophenyl)-*N*-methyl-*N*-(1-methylpropyl)-3-isoquinolinecarboxamide (PK11195) (K_d_ = 2.1–28 nM, see Figure S1) [7,30,31] (mTSPO_NMR_monomer in this study, PDBiD: 2MGY). Unfortunately, this model [20] shows some inconsistencies. These are likely to be caused, at least in part, by intrinsic limitations of the solution NMR structural template [29]. Indeed, the protein’s conformation may be affected by the use of ionic detergents (decylphosphocholine micelles) during the purification and the NMR measurements [28,32]. This concern is corroborated by binding assays of PK11195 in ionic detergents [30,32]: the affinity is abolished by purifying the protein in a sodium dodecyl sulfate detergent, and it is restored up to about 1.5 nM after the removal of SDS and reconstruction into liposomes [30]. Hence, the proposed solution NMR monomer structure may be in a conformational state different from the one in the mitochondrial membrane [28].

Prompted by the pharmacological importance of the protein, here we have constructed a new model of dimeric mTSPO. We use as a template the prokaryotic protein X-ray structure, which features the same fold as the eukaryotic one [34]. Our prediction turns out to be fully consistent with the available experimental topological information, thus delving for the first time into the structural determinants of this exceptionally important target for pharmacological interventions and diagnostics.

## 2. Results

**Figure 2:**
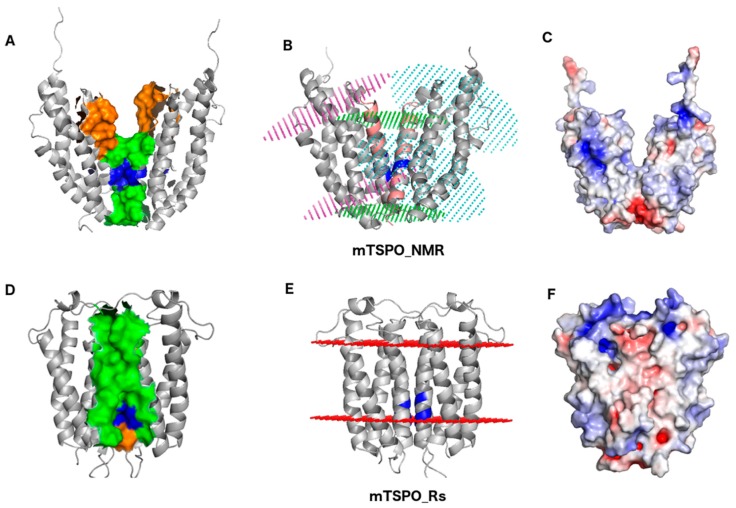
The mTSPO_NMR (**A**–**C**) and mTSPO_Rs dimers (**D**–**F**). (**A**) The G83XXXG87 motif (blue surface) of mTSPO_NMR is located at the dimer interface, consistently with the experimental findings [20] (Table 1). Other residues participating to the dimer interface in the model and according to experiment [20] are represented by green and orange surfaces, respectively. (**B**) Embedding of mTSPO_NMR in a bilayer membrane according to the PPM server [35]. The membrane boundaries for the individual monomers are shown by pink and cyan spheres. Glycophorin A (PDBiD: 5EH4, shown as pink ribbons) has been used as template to build the dimer, following the procedure of [20]. The membrane boundaries of this protein derived from the Orientation of Proteins in Membranes (OPM) database [35] are shown as green spheres. The membrane boundaries of mTSPO_NMR are not parallel to those of the template guiding the dimerization. (**C**) mTSPO_NMR electrostatic surface potential. The surface potentials are calculated using APBS [37]. Red and blue surfaces represent negative and positive electrostatic potentials (−5 kT/e, +5 kT/e), respectively. The exposed positively charged (blue) surface in are generated by R27, R46, R156, and R161. (**D**) The blue surface shows the G83XXXG87 motif of mTSPO_Rs. The green surface shows the other residues participating to the dimer interface in each monomer. The orange surface shows the residues participating to the dimer interface according to the experimental assignments data [20] not already included in the previous selections. (**E**) Embedding of mTSPO_Rs in a bilayer membrane according to the PPM server calculations. The membrane boundaries are shown in red. (**F**) mTSPO_Rs electrostatic surface potential. These residues that are exposed towards the lipid membrane in **C**, have now the sidechains oriented towards the cytoplasm.

The only model reported so far for the functional, dimeric form of eukaryotic TSPOs (mTSPO_NMR hereafter) is based on mTSPO_NMR_monomer structure [29]. As mTSPO_NMR’s 3D coordinates are currently not available, we have repeated the procedure reported in [20] to construct and analyze it. The mTSPO_NMR model (Figure 2A) reveals some drawbacks: (i) Residues N92, W93, W95, I98, F100, G101, and A102, experimentally identified to be located at the monomer-monomer interface, are separated by a distance of 9 Å or more (Table 1 and Figure 2A); (ii) Conversely, there are six residues, from F74 to M79, located at the dimer interface, that were found elsewhere experimentally (Table 1 and Figure 2A); (iii) The predicted embedding of the protein into the membrane (as obtained with the ‘Positioning of Proteins in Membranes’, PPM, server [35]) shows that the dimer is significantly tilted with respect to the membrane plane. As a result, the thickness of the hydrophobic region of this model is far smaller (14 Å) than that required in a biological hydrophobic membrane region (Figure 2B and Table S2). Therefore, it is unlikely that mTSPO_NMR can be correctly embedded in a biological membrane. We underline that the embedding in the mitochondria membrane is compatible with the generalized membrane used by the PPM server (http://opm.phar.umich.edu/protein.php?search=tspo). Indeed, the PPM database contains the monomer of mouse TSPO embedded in mitochondria membrane and either the tilt and membrane thickness is fully compatible with our modeled structure (Figure 2E); (iv) Several charged residues are exposed towards the membrane (see Figure 2C); (v) PK11195 forms highly stabilizing interactions in the *Bacillus cereus* TSPO/PK11195 X-ray structure (PDBiD: 4RYI) (these are π-stacking interactions with F90 and hydrogen bonds (H-bonds) with W51 (Figure S2B,C)) [36], not present in this model. The ligand forms here mostly hydrophobic interactions with the protein (Figure S2A). In particular, the two residues corresponding in the sequence alignment to F90 and W51 of the prokaryotic protein (W95 and W53) are not oriented in an optimal way inside the binding pocket (Figure S2), thus impeding a similar interaction pattern. This finding is not consistent with the experimental evidence that PK11195 binds stronger to mammalian TSPOs [7,30,31] than to prokaryotic ones [32].

We conclude that these inconsistencies with experimental data may limit the predictive power of this model. Part of these drawbacks might be caused by limitations of the template structure used.

Therefore, we decided to build a new model (mTSPO_Rs hereafter), based on homology modeling using the corresponding prokaryotic dimer from RsTSPO, for which an X-ray structure is available (PDBiD: 4UC1) and whose folding is comparable with the ones of the eukaryotic protein [34]. This template appears rather suitable for this prediction.

Most importantly, our prediction turns out to be consistent with the results from solid-state NMR (Figure 2). The two subunits of mTSPO_Rs arrange very differently than those of mTSPO_NMR: they are oriented almost perpendicularly to the membrane bilayer. As a result, the thickness of the hydrophobic region (~22 Å, see Table S2 and Figure 2D,E) is compatible with that of other membrane proteins [35]. Therefore, mTSPO_Rs can be embedded into a membrane bilayer. The side chains of the charged residues point toward the cytosolic side, as expected for a membrane protein (Figure 2F). This observation is in line with [34], which compares the electrostatic surface of RsTSPO with that of mTSPO_NMR_monomer. The dimer interface is mainly hydrophobic (Table 1). It extends along 33 Å. It involves (i) V80, G83, W93, I98, and A102 from TM-III and V118 from TM-IV, consistently with experimental data [20] (Table 1); (ii) the G83XXXG87 motif, also in agreement with experimental data [20]; and (iii) the W95XPXF99 motif. Here, the side chains of P97 and I98 are directed to the interface, while the side chains of W95, P96, and F99 point towards the inner binding cavity of the protein. This is consistent with the experimental findings that assigns to the dimer interface residues W95 and I98 from this motif [20]. The other residues occurring at the dimer interface in the model, are placed in the proximity of other residues identified experimentally. Thus, we cannot exclude that small structural adjustments in the area of the interface could optimize the interactions, yielding monomer-monomer contacts fully consistent with the experimental assignments.

The topology of the monomers in the proposed model presents few, still significant differences with respect the ones in mTSPO_NMR (Figure 1B) in the dispositions of the transmembrane and loops regions. In turn, the position of several residues located in the binding site differ markedly from those of mTSPO_NMR (Figure S3): W95, F99, and F100 orient towards the binding cavity, creating an aromatic pocket suitable to accommodate the PK11195 ligand and offering multiple potential anchor points for π-stacking interactions. Within the well-known limitations associated with the docking procedures to a homology model [38], the induced-fit docking [39,40] of PK11195 using Glide [41,42,43] and MOE [44,45] scoring functions, confirms that this is the case. Indeed, the ligand establishes at least one π-stacking and one H-bond in each of the binding sites. The residues involved in these interactions are W53, W93, and P96 (Figure S2D,E). As expected, in both subunits the ligand shares the same binding pose, and only small differences can be observed in the two binding pockets. The interactions patterns are not too different from those found in BcTSPO/PK11195 X-tray structure [36]. With the same approach, the binding modes of other known mammalian TSPO tracers in mTSPO_Rs have been also predicted (see Table S7 of the Supporting Information). The protein-ligand interactions turn out to be overall similar to those discussed for PK11195.

We analyzed the coevolution among relevant regions of the protein (Table 1 and Figure S6). W95 from the W95XP97XF99 motif, and N151, belonging to the cholesterol recognition amino acid consensus (CRAC) motif (residues 149–156) [20,46,47] have coevolved. Interestingly, the W95XP97XF99 motif has also coevolved with binding site residues in LP-I and TM-II residues. Moreover, CRAC domain residue L150 has coevolved with residues of the binding region. These include P40, P44, and T48, placed in LP-I, as well as W53 placed in TM-II. Furthermore, residues Y140 and L144 belonging to the mirror cholesterol recognition amino acid consensus motif (CARC) [47] have coevolved with binding-site residues of TM-II (W53, Y57). Our model shows that the W95XP97XF99 motif is involved in ligand binding and receptor dimerization. Our coevolution analysis accordingly suggest that W95XP97XF99 is evolutionary linked to other binding sites residues, as well as with the cholesterol-binding motif. This is in line with the suggested role of cholesterol in regulating TSPO dimerization [20]. Notably, the W95XP97XF99 motif is located at the dimer interface only in our model.

We conclude that our prediction is overall in good agreement with experimental data and appears a reasonable model of the functional form of mTSPO. In the following section, we discuss the feasibility of the template structures used for the structural predictions discussed above.

### Analysis of the Templates for mTSPO_NMR and for mTSPO_Rs

**Figure 3:**
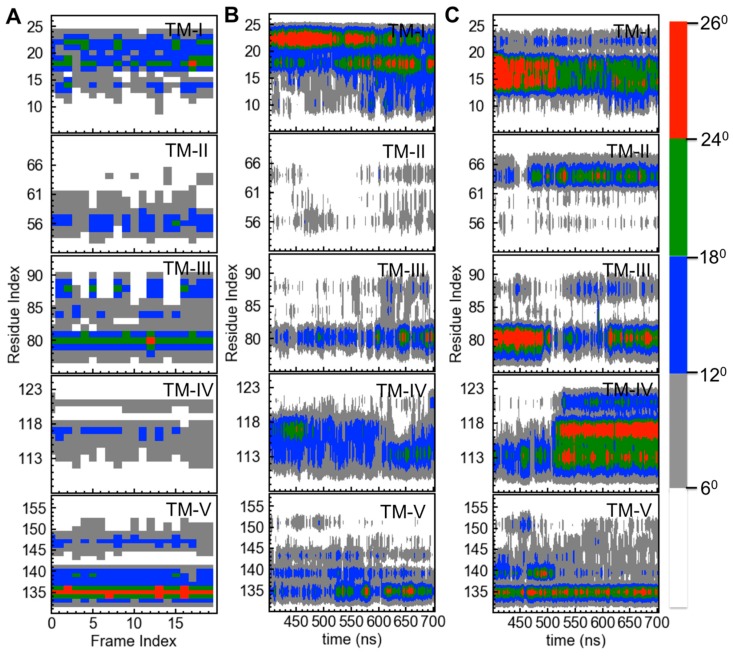
The helix bending in mTSPO_NMR_monomer. The values are either reported for the deposited 20 NMR structures of mTSPO_NMR_monomer (PDBiD: 2MGY) (**A**), for mTSPO_NMR_monomer without (**B**) or with PK11195 (**C**) as a function of the simulation time. Only the last 300 ns are shown. The Figure was made using the “bendix” plugin of the VMD program [48].

**Figure 4:**
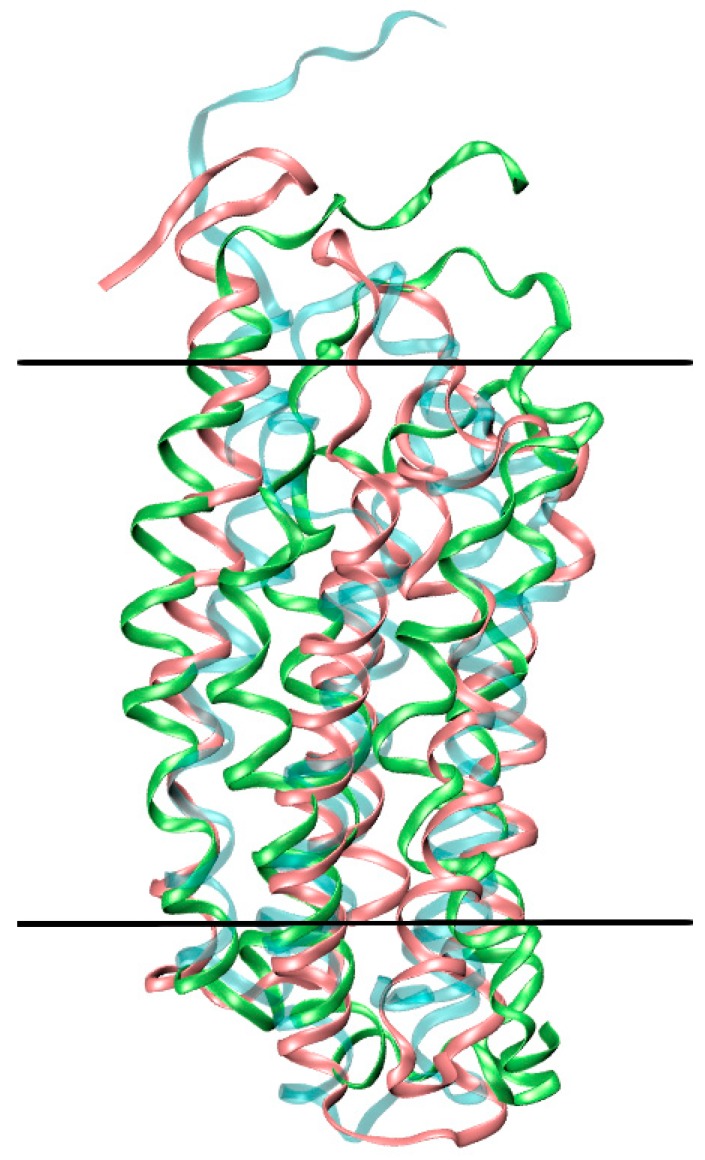
Distortion of mTSPO_NMR_monomer in the lipid bilayer. The initial NMR structure (cyan) is superimposed to mTSPO_NMR_monomer in complex with PK11195 (green) and mTSPO_NMR_monomer without ligand (pink) after MD equilibration. The boundaries of the membrane are approximately shown as black lines.

The ionic detergents used to determine the mTSPO_NMR_monomer structure [29] may affect significantly the structure of the receptor. Indeed, these modify the binding affinity of TSPO for PK11195 [30,32], possibly because of structural changes of the receptor. 700-ns MD simulations of mTSPO_NMR_monomer with and without ligand, in explicit solvent and embedded in a membrane environment (see Methods for details) suggest that this is indeed the case. The obtained conformations appeared to be equilibrated after 400 ns and they lead to a different conformation compared with the NMR structure (See SI). While the ligand mostly retains its binding pose (see SI), the bending of the helices (particularly of TM-II and TM-IV, Figure 3) increases, especially in the presence of the ligand. This leads to a distortion of the helix bundle (Figure 4) and to a shrinking of the protein along the direction perpendicular to the membrane, especially in the holo protein.

Moreover, we have analyzed the membrane embedding of the monomers that build our new mTSPO_Rs dimer and compared it to the embedding of the mTSPO_NMR_monomer structure (PDBiD: 2MGY). Using the QMEANBrane program [49], we observe that the monomer of our model (mTSPO_Rs_monomer) shows higher scores than the NMR monomeric structure, indicating a better membrane embedding already at the monomeric level (Figure S4).

All the multimeric TSPO structures are either dimers or trimers of RsTSPO from *Rhodobacter sphaeroides* and BcTSPO from *Bacillus cereus* (Table S1). They have a sequence identity with mTSPO is of 32% and 27%, respectively [28]. These proteins (i) have been crystallized in lipidic cubic phase conditions [34,50] which have proven to be a reliable method to determine X-ray structures of membrane proteins; (ii) they present a correctly folded membrane protein according to the QMEANBrane server, which takes into account general statistical characteristics of membrane proteins, but not membrane composition [49]; and (iii) they feature a characteristic motif located at the subunit/subunit interface (A75XXXA79 in TM-III of RsTSPO and G44XXXG48 in TM-II of BcTSPO). Both motifs are involved in the stabilization of helix-helix contacts across different transmembrane proteins [51]. The alignment of RsTSPO and mTSPO sequences (Table S1) shows that the A75XXXA79 motif matches the G83XXXG87 motif of mTSPO—as a crucial element in the dimerization of mTSPO [20]—and in the resulting model the latest is correctly located at the dimer interface. This is not the case for the G44XXXG48 motif, which does not align with the G83XXXG87 motif of mTSPO and in the resulting model it would not be located at the dimer interface. Thus, BcTSPO, by lacking this crucial feature, appears not to be a suitable template for a structural prediction of the mTSPO functional dimer.

Although it shares a modest sequence identity with the mouse protein [28], all the results reported above leads us to suggest that RsTSPO represents a fairly good template for our structural predictions.

## 3. Discussion

In this paper we propose an alternative dimer model of mammalian TSPO based on the deposited X-ray structure of bacterial RsTSPO (mTSPO_Rs) with respect the one based on the NMR structure of mTSPO (mTSPO_NMR). The major differences can be appreciated from Figure 2 and they regard (i) the dimer interface, (ii) the embedding in a membrane bilayer, and (iii) the binding pocket and the effect on ligand binding. For all of these aspects, the mTSPO_Rs dimer model appears more suitable than mTSPO_NMR to describe the physiologically-relevant dimeric structure. The residues at the dimer interface in mTSPO_Rs match those identified experimentally in solid state NMR [20], the simulated embedding of mTSPO_Rs in a membrane is reasonable, and the predicted binding of the prototypical ligand PK11195 if favored in mTSPO_Rs with respect to mTSPO_NMR. Remarkably, our proposed model can rationalize the higher affinity of PK11195 for mammalian TSPO with respect the bacterial protein. This is not the case for mTSPO_NMR, where the type and number of interactions do not seem consistent with the measured nanomolar affinity [7,30,31].

Our model also shows that the W95XP97XF99 motif is involved in both ligand binding and dimerization. Accordingly, we found that W95XP97XF99 residues have coevolved with residues responsible for ligand binding and with the ones in the cholesterol binding motifs. This is in agreement with the suggested role of cholesterol binding on the monomer-dimer equilibrium [20]. Furthermore, the combined molecular modeling and co-evolution analyses suggest a possible interplay between the W95XP97XF99 dimerization motif and cholesterol and ligand binding during evolution. Notably this motif is not part of the dimer interface in mTSPO_NMR, while it is in mTSPO_Rs.

In conclusion, our model is in agreement with the available experimental data and can explain several aspects of TSPO features related to dimerization and cholesterol binding.

## 4. Materials and Methods

### 4.1. Bioinformatics Analyses

We used the ConSurf server (http://consurf.tau.ac.il/2016/) [33] to perform a conservation analysis across TSPO homologous sequences of TSPO. We set a cut-off value of 95% of sequence identity and minimal cut off value of 35% homologue identity to retrieve the homologous sequences to the mTSPO sequence. Further obtained multiple sequence alignment visualized online (http://molsim.sci.univr.it/TSPO/).

The mTSPO sequence was submitted to the HHPRED server [52,53] to identify the most suitable template. The Modeller program [54] then generated multiple homology models of the monomer based on the template. We used a very slow mode of MD annealing technique for precise refinements. We selected the best homology models according to DOPE [55] and GA341 scores [56], as well as the model quality according to the QMEANBrane module from Swiss-model [49] (Figure S4).

We cross checked our alignment and model by performing an extensive conservation analysis to identify the evolutionary relevance of the different amino acid positions in the protein, based on the phylogenetic relation between homologous sequences to the mTSPO. A total of 1940 sequences were used to build the Multiple Sequence Alignment, 1882 of which are unique and 148 are homologous sequences to the mTSPO (visualized online http://molsim.sci.univr.it/TSPO/). As discussed in the main text, we selected RsTSPO (PDBiD: 4UC1) as a template to build dimer for mTSPO. The homology model for the dimer has been generated using Modeller [54] and validated similarly as monomer homology model.

CoeViz, a web-based tool [57], is used to identify the evolutionary coupling among the residues and the functionally related residues in mTSPO. Evolutionary coupling residue pairs were selected based on their pairwise χ^2^ scores [58] (cutoff value > 0.7, See Table 1 and Figure S6).

The Positioning of Proteins in Membranes (PPM) server [35] assigns spatial positions of molecules inside membranes by optimizing their transfer energy from water to the lipid bilayer [59]. It was used here to assess the embedding of each studied system in the membrane bilayer. Molecular graphics were drawn using Pymol [60] and VMD [61].

The accession numbers (PDBiD) of the proteins relevant for this work are: 2MGY, 4UC1, 4RYI, and 5EH4.

The 3D structures of the dimer models of mTSPO discussed in this work are available upon request.

### 4.2. MD Simulations of mTSPO_NMR_monomer

The first frame of the NMR ensemble (PDBiD 2MGY) is embedded in a membrane composed by phosphatidylcholine (POPC, 31%), phosphatidylethanolamine (POPE, 41%) and cholesterol (CHL, 28%) and enclosed in a water box with dimensions (nm) 10.1 × 10.1 × 12.8. The total charge of this system is kept neutral and the ionic strength is set to 0.15 M by adding potassium (K^+^) and chloride (Cl^−^) ions. The whole system includes mTSPO, PK11195, POPC (84 molecules), POPE (116 molecules), CHL (98 molecules), water (27126 molecules), K^+^ (118 ions), and Cl^−^ (124 ions). In the simulation of the apo protein, only the ligand PK11195 is removed.

The AMBER99SB-ILDN force fields [62], the Slipids [63,64], and TIP3P [65] were used for the protein and ions, the lipids, and water, respectively. The General Amber force field (GAFF) parameters [66] were used for PK11195, along with the RESP atomic charge [67,68] fitted with the electrostatic potential (ESP) from Gaussian 09 [69] calculation with the HF-6-31G* basis set [70,71]. The topology of PK11195 was then converted to GROMACS format using the ACPYPE tool [72].

The Particle Mesh Ewald method [73] was used to treat the long-range electrostatic interaction with a real space cutoff of 1.2 nm. A 1.2 nm cutoff was also used for the short-range non-bonded interaction. A time step of 2 fs was set. The LINCS algorithm [74] was applied to constrain all bonds involving hydrogen atoms. Constant temperature and pressure conditions were achieved via independently coupling protein/PK11195, lipids, solvent, and ions to a Nosè-Hoover thermostat [75] at 315 K and Parrinello-Rahman barostat [76] at 1 atm. For each simulation, the systems underwent a two-steps minimization without restraints. The minimization with conjugated gradient method [77] followed the steepest descent method. Then 1-ns simulated annealing and 10-ns equilibration with positional restraint using a force constant of 1000 kJ∙mol^-1^∙nm^−2^ on the heavy atoms of the protein were carried out. The last 700-ns MD at 315 K and 1 atm was performed with frames collected every 20 ps.

The root mean square deviation of backbone atoms (N, C_α_, C atoms, bb-RMSD) and the root mean square fluctuation (RMSF) were calculated using the g_rms and g_rmsf codes from the Gromacs package [78]. The 13th structure of the mTSPO NMR ensemble (PDBiD: 2MGY) was selected as the reference. This choice was based on the results of pairwise RMSD analysis, hydrogen bonds count, and secondary structure analysis performed on the twenty structures of NMR ensemble. The secondary structure was defined and calculated with do_dssp [79] embedded in Gromacs. The distortion of helix was evaluated with the Bendix [48] plug-in in VMD software [61], using default parameters. The g_cluster module with gromos method in Gromacs [80] was used to perform cluster analysis on the converged part of the MD simulation (after 400 ns). The cutoff based on bb-RMSD for clustering is set to 1.2 Å. From the cluster analysis, the central frame of the most populated cluster is selected as representative structure of mTSPO_NMR_monomer after MD equilibration for the holo and apo proteins simulated. The trj_cavity package [81] within GROMACS was selected to calculate the volume of the binding cavity, using a grid spacing of 1.4 Å. The number of H-bonds was calculated with g_hbond in GROMACS. The PAD flexibility index [82], the distribution of geometry center, dihedral angles, rotation angle of PK11195 and contact percentage between PK11195 and residues are all calculated with an in-house code. The contact percentage is defined as the minimum distance between any atoms of side chains and ligand, both excluding hydrogen atoms, less than 4.0 Å. Water molecule with distance less than 5 Å from the geometric center is accounted as molecules within the binding pocket. MD simulations were performed using Gromacs package [78] on supercomputer.

## 5. Conclusions

Here, we have presented a computational study aiming to shed light on the structural determinants of the functional form of mammalian TSPO. The reliability of the dimer construct based on the NMR monomer of the mouse protein ([20] and this work) appears weakened by some inconsistencies, possibly caused by the fact that the NMR experiments were carried out under conditions far from the physiological ones. Therefore, we have proposed an alternative model, quite different from the previous one, which is in good agreement with the available experimental findings and supports dimer formation. This model may foster pharmacological studies aimed at a rational design of novel TSPO targeting ligands.

## Figures and Tables

**Table 1:** Residues of the mTSPO/PK11195 complex located either in the protein binding cavities or at the dimer interface, according to the experimental data [20] and to the proposed dimer models. These models are based either on the NMR structure (mTSPO_NMR, [20] and this work) or on the protein from *Rhodobacter Sphaeroides* (RsTSPO, this work). Residues at the interface accordingly to both the NMR data [20] and mTSPO_Rs are in bold. mTSPO_Rs’ binding pockets do not include exactly the same residues because of differences of the ligands’ binding poses, as obtained with docking simulations described in the text. On the contrary, mTSPO_NMR binding pockets include by construction exactly the same residues. The residues within 5 Å from the ligand are assigned to the binding cavity and reported in this table. The corresponding list of the residues within 4 Å and 6 Å from the ligand is shown in Table S6 and Figure S5. We report also a list of the residues with a conservation rate higher than 85% in multiple sequence alignment across 148 homologous sequences to the mTSPO, as determined with the ConSurf server [33], along with the topological area of the protein where these sequences are found. A detailed discussion of the evolutionary coupling of the residues is presented in the Results section and in Figure S6.

Region	Inferred by Experiment	mTSPO_NMR		mTSPO_Rs	
**Binding Cavity**	G19, A23, V26, R27, H43, R46, L49, A50, I52, W53, W95, W107, A110, D111, L114, W143, A147, L150, N151	Subunit A	Subunit B	Subunit A	Subunit B
		G18, G19, F20, G22, A23, V26, R27, G30, L31, K39, P40, S41, H43, P44, P45, R46, L49, A50, I52, W53, W93, W95, W107, A108, A110, D111, L114, W143, F146, A147, T148, L150, N151	G18, G19, F20, G22, A23, V26, R27, G30, L31, K39, P40, S41, H43, P44, P45, R46, L49, A50, I52, W53, W93, W95, W107, A108, A110, D111, L114, W143, F146, A147, T148, L150, N151	P15, G18, G19, M21, G22, A23, F25, V26, R27, G28, E29, Y34, K39, H43, P44, R46, L49, A50, W53, G54, Y57, N92, W93, W95, P96, F99, F100, L112, W143, F146, A147, T148, L150, N151, V154	G18, M21, G22, A23, F25, Y34, H43, P44, R46, L49, A50, W53, L56, Y57, N92, W93, A9, W95, P96, P97, F99, F100, L112, V115, Y140, L141, W143, A147, L150
**Dimer Interface**	**V80**, **G83**, Q88, N92, **W93**, W95, **I98**, F100, G101, **A102**, **D111**, **V118**	F74, T75, E76, D77, M79, **V80**, P81, **G83**, L84, T86, G87, Q88, A90, L91		V6, P7, G10, L11, L13, V14, L17, G18, F20, M21, Y24 V26, R27(A) M79, **V80**, L82, **G83**, L84, Y85, T86, G87, A90, L91, **W93**, A94, P97, **I98**, **A102**, Q104, W107, A108, A110, **D111**, L114, **V118**, A121, A125	
**Conserved Residues**	**LP-I**: L37, P40, P44, P45, **TM-II**: W53, L56, G61, **TM-III**: N92, W95, F99, F100, **TM-V**: L136, P139, Y140, W143, A147, L150, N151				
**Evolutionary Coupling**	P40, P45 coevolve with L150; P44, P45 with W95; W53 with L56, A147, L150; W95 with A147 and N151				

## Supplementary Materials

Supplementary materials can be found at http://www.mdpi.com/1422-0067/19/9/2588/s1.

## Abbreviations

TSPO	Translocator membrane protein
PET	Positron emission tomography
RsTSPO	*Rhodobacter Sphaeroides*
BcTSPO	*Bacillus Cereus*
mTSPO_NMR_monomer	Monomer of mTSPO solved by solution NMR experiment, PDBiD: 2MGY
mTSPO_NMR	Dimer model of mTSPO. The prediction is based on mTSPO_NMR_monomer structure
mTSPO_Rs	Dimer model of mTSPO. The prediction is based on the RsTSPO structure
MD	Molecular dynamics
bb-RMSD	Root-mean square deviation of backbone atoms (N, C_α_, C atoms)
RMSF	Root-mean square fluctuation
