# Supplementary material for: Structural Prediction of the Dimeric Form of the Mammalian Translocator Membrane Protein TSPO: A Key Target for Brain Diagnostics
Source: Int J Mol Sci. 2018 Aug 31;19(9):2588. doi: 10.3390/ijms19092588 (PMC6165245; doi:10.3390/ijms19092588)
Supplement: Supplementary file 1 [file ijms-19-02588-s001.pdf]

Supplementary Information

Structural Prediction of the Dimeric Form of the Mammalian Translocator Membrane Protein TSPO: A Key Target for Brain Diagnostics

Juan Zeng[1,2*], Riccardo Guareschi[1*], Mangesh Damre[3,4*], Ruyin Cao[1], Achim Kless[5], Bernd Neumaier[6], Andreas Bauer[7], Alejandro Giorgetti[1,3**], Paolo Carloni[1,8], Giulia Rossetti[1,9,10 **]

1. Institute for Advanced Simulations (IAS)-5 / Institute for Neuroscience and Medicine (INM)-9, Forschungszentrum Jülich, Germany
2. Laboratory of Computational Chemistry and Drug Design, Laboratory of Chemical Genomics, Peking University Shenzhen Graduate School, Shenzhen 518055, China
3. Department of Biotechnology, University of Verona, Italy
4. Neurobiology, International School for Advanced Studies (SISSA), Trieste (Italy)
5. Grünenthal Innovation, Translational Science & Intelligence, Grünenthal GmbH, 52078 Aachen, Germany
6. Institute for Neuroscience and Medicine (INM)-5, Forschungszentrum Jülich, Germany
7. Institute for Neuroscience and Medicine (INM)-2, Forschungszentrum Jülich, Germany
8. RWTH Aachen University, Aachen, Germany
9. Jülich Supercomputing Center (JSC), Forschungszentrum Jülich, Germany
10. University Hospital Aachen, RWTH Aachen University, Germany

*Equally contributes; **corresponding author: g.rossetti@fz-juelich.de; alejandro.giorgetti@univr.it

1. Bioinformatics Analysis

```
Q9RFC8  RsTSPO  1     MNMDWALFLTFLAACGAPATTGA---LLKPDEWYDNLNKPWWNPPRWVFPLAWTSLYFLM
 57
P50637  mTSPO   1     MPESWVPAVGLTLVPSLGGFMGAYFVRGEGLRWYASLQKPSWHPPRWTLAPIWGTLYSAM
 60
Q81BL7  BcTSPO  1     MFMKKSSIIVFFLTYG--LFYVSSVLFPIDRTWYDALEKPSWTPPGMTIGMIWAVLFGLI     58
                      *  .  : : . .    :      **  *:** * **  .:  *  *: :

Q9RFC8  RsTSPO  58    SLAAMRVAQL-----EGSGQALAFYAAQLAFNTLWTPVFFGMKRMATALAVVMVMWLFVA
 112
P50637  mTSPO   61    GYGSYIVWKELGGFTEDAMVPLGLYTGQLALNWAWPPIFFGARQMGWALADLLLVSGVAT
 120
Q81BL7  BcTSPO  59    ALSVAIIYNNYGFKPK-T--FWFLFLLNYIFNQAFSYFQFSQKNLFLATVDCLLVAITTL     115
                      . .  : :    : :   :: :  :* :  . *. .:: *  .  :::   .

Q9RFC8  RsTSPO  113   ATMWAFFQLDTWAGVLFVPYLIWATAATGLNFEAMRLNWNRPEARA---     158
P50637  mTSPO   121   ATTLAWHRVSPPAARLLYPYLAWLAFATVLNYYVWRDNSGRRGGSRLPE     169
Q81BL7  BcTSPO  116   LLIMFSSNLSKVSAWLLIPYFLWSAFATYLSWTIYSIN----------     153
                          .:.  :. *: **: * : ** *.:     *
```

Table S1. Multiple sequence alignment across mTSPO, RsTSPO, and BcTSPO, as obtained with the ClustalOmega tool [2]. Residues belonging to the transmembrane region are colored in red. Positions which have a single, fully-conserved residue are indicated by **'*'.** The conservation between groups of strongly similar properties—scoring > 0.5 in the Gonnet PAM 250 matrix [4]—are indicated by **':'** (colon). The conservation between groups of weakly similar properties—scoring =< 0.5 in the Gonnet PAM 250 matrix [4]—are indicated by **'.'** (period).

A) Protein Orientation in Membrane			
	**mTSPO_NMR_monomer (PDBiD: 2MGY)**	**mTSPO_NMR**	**mTSPO_Rs**
Depth/Hydrophobic Thickness (Å)	28.6 ± 1.6	14.6 ± 1.2	21.8 ± 3.6
Tilt angle (°) dimer	-	70°± 0°	2°± 2°
Tilt angle (°) monomer A	12° ± 1°	25°±0°	14°±0°
Tilt angle (°) monomer B	-	44°±0°	14°±0°

B) Membrane Embedding					
	**mTSPO_NMR_monomer (PDBiD: 2MGY)**	**mTSPO_NMR**		**mTSPO_RS**	
Subunit		**A**	**B**	**A**	**B**
Segments/ embedded residues	7-26, 46-63, 82-102, 106-124, 134-153	7-18, 20-21, 47, 51-68, 70-71, 76-90, 118, 121-139	6-17, 19, 42-57, 60, 79-93, 111-125, 139-150	8-23, 42, 44, 47-62, 83-98, 107-123, 126, 135-152	8-23, 42, 44, 47-62, 83-98, 107-123, 126, 136-153, 155
Transmembrane helices (residue interval)	I (7- 26), II(46- 63), III (82- 102), IV(106-124), V (134- 153)	Not recognized	Not recognized	I(8-23), II(48-62), III(83-98), IV(107-123), V(136-152)	I(8-23), II(48-62), III(83-98), IV(107-123), V(136-152)

Table S2. (**A**) Orientation of the proteins in the membrane is evaluated with the hydrophobic thickness and the tilt angle computed with the PPM server [5,6]. The hydrophobic thickness indicates the calculated maximal penetration depth of the protein in the lipid hydrophobic core. The tilt angle is the angle between the axis normal to the membrane's surface and the axis of each studied system. The errors for the evaluation of the hydrophobic thickness and the tilt angle correspond to fluctuations of 1 kcal/mol in the energy of the embedded protein [5,6]. (**B**) Membrane embedding. The table consists of two parts: a list of residues penetrating into the hydrocarbon core of the lipid bilayer for each subunit and parts of transmembrane alpha-helices or embedded into the hydrocarbon core [5,6].

```
Q ss_pred           CCCchHHHHHHHHHHHHHHHHHHHHhcccchHHHHhcCCCCCCCChhHHHHHHHHHHHHHHHHHHHHHHH
Q P50637            1          MPESWVPAVGLTLVPSLGGFMGAYFVRGEGLRWYASLQKPSWHPPRWTLAPIWGTLYSAMGYGSYIVWKE
70 (169)
Q Consensus         1          ~~~~~~~~~~~~~~~~~~~~~~~~~~~~~~~~wy~~l~~p~~~Pp~~~F~~iW~~iy~l~~~a~~~v~~~  70 (169)
                               |+....+......+|...+..++..   .+.+||++++||.++||+++|.++|.++|.+++++.++++++
T Consensus         1          ~~~~~~~~~~~~~~~~~~~~~~~~~---~~~~wy~~l~~P~~~Pp~~~f~~iW~viy~~~g~a~~~v~~~  67 (157)
T 4UC1              1          MNMDWALFLTFLAACGAPATTGALL---
KPDEWYDNLNKPWWNPPRWVFPLAWTSLYFLMSLAAMRVAQL  67 (157)
T ss_dssp           -CCCHHHHHHHHHHTHHHHGGGGGC---CCCCGGGGGGSCTTCCCTTHHHHHHHHHHHHHHHHHHHHHTS
T ss_pred           CcccHHHHHHHHHHHHHHHHHHHHc---CchHHHHhcCCCCCCchhHHHHHHHHHHHHHHHHHHHHHHhc

Q ss_pred           cCCCCcccHHHHHHHHHHHHHHHHHHHHHHhcchhHHHHHHHHHHHHHHHHHHHHHHhccCHHHHHHHHHH
Q P50637            71         LGGFTEDAMVPLGLYTGQLALNWAWPPIFFGARQMGWALADLLLVSGVATATTLAWHRVSPPAARLLYPY
140 (169)
Q Consensus         71         ~~~~~~~~~~~~~~~~~~~~l~~~W~~~ff~~~~~~~~a~~~~~~~~~~~~~~~~~~~~~~~~~~~~~a~~ll~py  140 (169)
                                  ++.+.....|.+++++|..|.+.+|+.+++..+++.++++.+++..++..+++.|+.++.++.||
T Consensus         68         -----~~~~~~~~~~~~~~~l~ln~~W~~~ff~~~~~~~~a~~~~~~~~~~~~~~~~~~~~~~~~~~~~a~~ll~Py  132 (157)
T 4UC1              68         -----EGSGQALAFYAAQLAFNTLWTPVFFGMKRMATALAVVMVMWLFVAATMWAFFQLDTWAGVLFVPY
132 (157)
T ss_dssp           -----TTCHHHHHHHHHHHHHHHHHHHHHHHHTSCCHHHHHHHHHHHHHHHHHHHHHHHHHHHHCHHHHHHHHHHH
T ss_pred           -----CCChhHHHHHHHHHHHHHHHHHHHHHHcCCcHHHHHHHHHHHHHHHHHHHHHHHHHHccChHHHHHHHHHH

Q ss_pred           HHHHHHHHHHHHHHHHHCCCCCCCC
Q P50637            141        LAWLAFATVLNYYVWRDNSGRRGGS  165 (169)
Q Consensus         141        ~~Wl~~at~ln~~~~~~~n~~~~~~~~~~  165 (169)
                               ++|+++++++++|...++.|.+...|.
T Consensus         133        l~Wl~~a~~~n~~~~~~~n~~~~~~~~~  157 (157)
T 4UC1              133        LIWATATTGLNFEAMRLNWNRPEAR  157 (157)
T ss_dssp           HHHHHHHHHHHHHHHHTC-------
T ss_pred           HHHHHHHHHHHHHHHHHcCCCcccC
```

Table S3. Target-template alignment obtained from the HHPRED server [7] . 4UC1_A translocator protein 18 kDa (TSPO); mitochondria, transport, 5 transmembrane helices; HET: PP9, P4C, OLC, Z0P; 1.8A Rhodobacter sphaeroides; Related PDB entries: 5DUO_C 5DUO_B 5DUO_A 4UC1_C 4UC1_B 4UC2_B 4UC2_A 4UC3_B 4UC3_A Probability: 99.94; E-value: 7.4E–28; score: 177.52; aligned cols: 157; identities: 32%; similarity: 0.538.

TSPO	PDBiD	No. of chains	Ligand	Method	Resolution (Å)
mTSPO*	2MGY	1	PK11195	Solution NMR	-
mTSPO	2N02	1	PK11195	Solution NMR	-
RsTSPO**	4UC1	3	Protoporphyrin IX	X-ray diffraction	1.8
RsTSPO	4UC2	2	-	X-ray diffraction	2.4
RsTSPO	4UC3	2	-	X-ray diffraction	2.5
RsTSPO	5DUO	3	Protoporphyrin IX	X-ray diffraction	2.4
BcTSPO***	4RYI	2	PK11195	X-ray diffraction	3.49
BcTSPO	4RYJ	2	-	X-ray diffraction	4.1
BcTSPO	4RYO	1	-	X-ray diffraction	1.6
BcTSPO	4RYQ	1	-	X-ray diffraction	1.7

BcTSPO	4RYN	1	-	X-ray diffraction	2.01
BcTSPO	4RYM	1	-	X-ray diffraction	2.8
BcTSPO	4RYR	1	-	X-ray diffraction	1.7

Table S4. TSPO deposited structures in the Protein Data Bank. * mTSPO = *Mus musculus* TSPO, ** RsTSPO = *Rhodobacter sphaeroides* TSPO, *** BcTSPO = *Bacillus cereus*

System	Acronym	Description
**1)**	mTSPO_NMR	Dimer model of mTSPO based on the NMR study reported in Ref. [8]
**2)**	mTSPO_NMR_monomer	Monomer of mTSPO studied with MD simulation and used to build **1)**. The coordinates are taken from the PDBiD: 2MGY[9]
**3)**	mTSPO_Rs	Dimer model of mTSPO built by us, based on the X-ray coordinates of prokaryotic RsTSPO [10]
**4)**	mTSPO_Rs_monomer	Monomer of the structure described in system **3)**
**5)**	BcTSPO	TSPO protein from the bacterium *Bacillus cereus* (PDBiD: 4RYI [11]), used to compare the binding of PK11195 with the binding of the same ligand observed in system **1)** and system **2)**
**6)**	RsTSPO	TSPO protein from the bacterium *Rhodobacter sphaeroides* (PDBiD: 4UC1 [10]), used to build system **3)**

Table S5. Acronyms and descriptions for the systems analyzed.

mTSPO_Rs Binding Site Residues			
Subunit A		Subunit B	
4 Å	6 Å	4 Å	6 Å
G18, M21, G22, F25, V26, R27, G28, E29, Y34, H43, P44, L49, A50, W53, Y57, N92, W95, P96, F99, F100, L112, W143, A147, L150, N151, V154	P15, L17, G18, G19, M21, G22, A23, Y24, F25, V26, R27, G28, E29, G30, Y34, L37, K39, P40, H43, P44, R46, L49, A50, W53, G54, Y57, N92, W93, W95, P96, F99, F100, L112, W143, F146, A147, T148, L150, N151, V154	G18, G22, Y34, R46, L49, A50, W53, L56, Y57, N92, W93, W95, P96, F99, F100, L112, V115, Y140, L141, A147, L150	L17, G18, G19, M21, G22, A23, F25, Y34, H43, P44, R46, W47, L49, A50, P51, W53, L56, Y57, M60, Q88, L89, L91, N92, W93, A94, W95, P96, P97, F99, F100, L112, V115, Y140, L141, W143, F146, A147, L150, N151
**mTSPO_NMR Binding Site Residues**			
**Subunit A**		**Subunit B**	
**4 Å**	**6 Å**	**4 Å**	**6 Å**
G19, A23, V26, R27, K39, S41, H43, P44, P45, R46, L49, A50, I52, W53, W95, W107, A108, A110, D111, L114, W143, F146, A147, L150, N151	P15, G18, G19, F20, G22, A23, Y24, F25, V26, R27, G30, L31, K39, P40, S41, H43, P44, P45, R46, W47, T48, L49, A50, I52, W53, W93, W95, P96, I98, A102, W107, A108, L109, A110, D111, L112, L114, W143, F146, A147, T148, L150, N151, Y152	G19, A23, V26, R27, K39, S41, H43, P44, P45, R46, L49, A50, I52, W53, W95, W107, A108, A110, D111, L114, W143, F146, A147, L150, N151	P15, G18, G19, F20, G22, A23, Y24, F25, V26, R27, G30, L31, K39, P40, S41, H43, P44, P45, R46, W47, T48, L49, A50, I52, W53, W93, W95, P96, I98, A102, W107, A108, L109, A110, D111, L112, L114, W143, F146, A147, T148, L150, N151, Y152

Table S6. List of the residues within 4 Å or 6 Å from the PK11195 ligand in mTSPO_Rs and mTSPO_NMR.

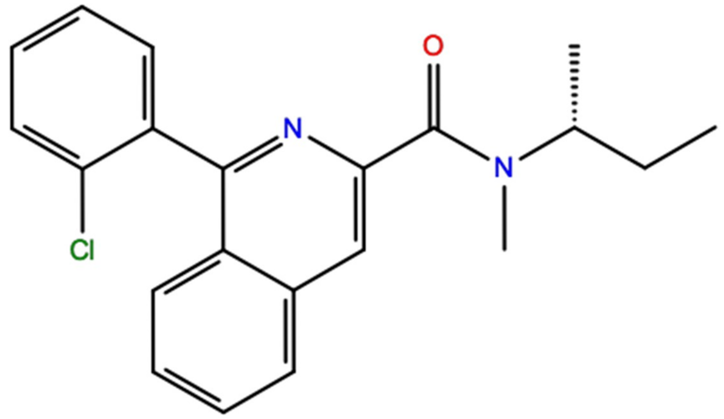

Figure S1. Chemical structure of 1-(2-chlorophenyl)-N-methyl-N-(1-methylpropyl)-3-isoquinolinecarboxamide (PK11195).

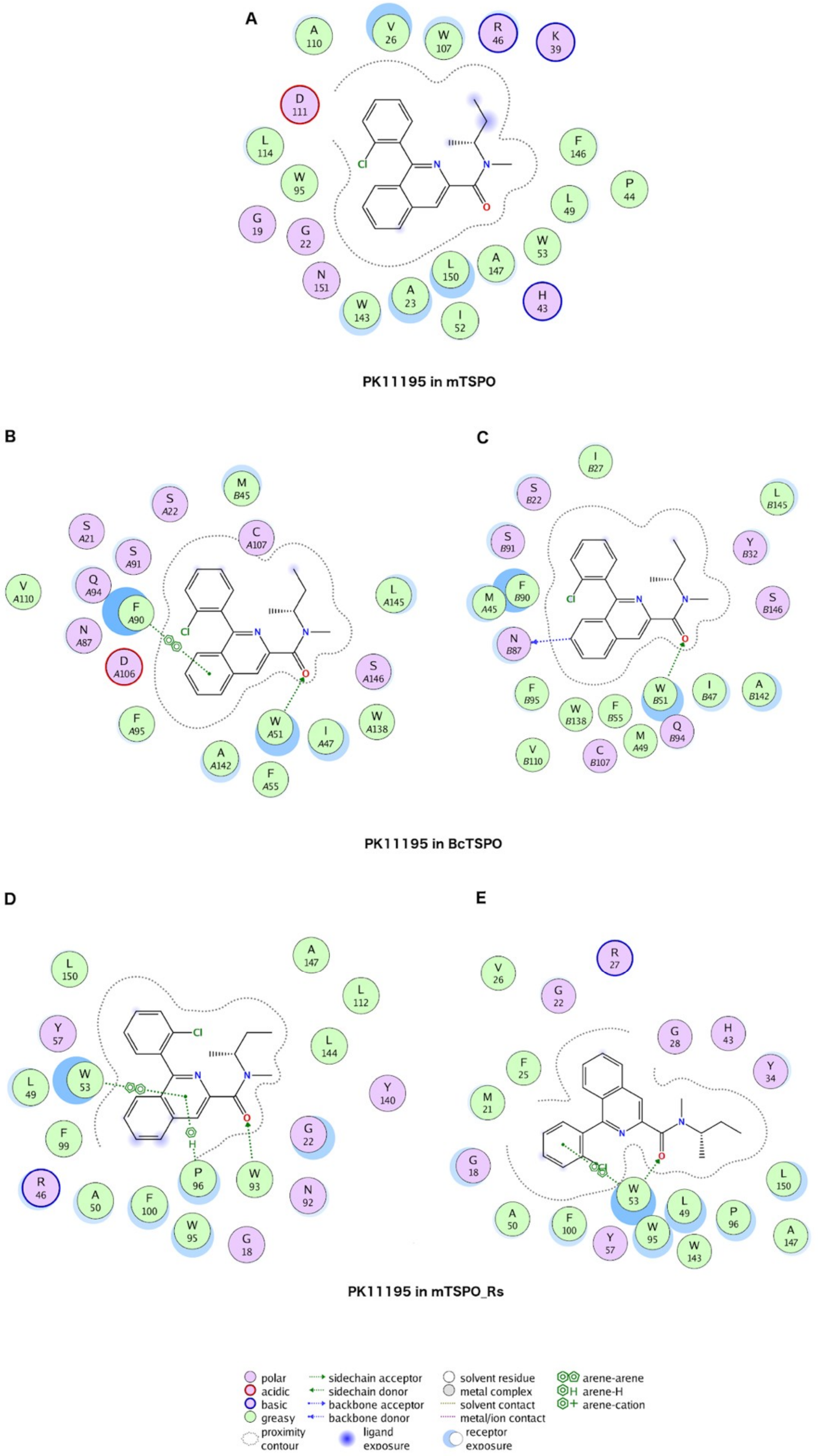

Figure S2. Binding pose of PK11195 in the binding pocket of mTSPO_NMR_monomer structure [9] (**A**), BcTSPO dimeric X-ray structure [11] (**B,C**) and mTSPO_Rs (**D-E**). In (**A**), only hydrophobic contacts are present. In (**B,C**), the two binding pockets are not identical. However, the ligand is stabilized in both cases by H-bond with W51. In one subunit, also a -stacking contact is detected with F90. In (**D,E**), the ligand establishes one H-bond with either W93 in one subunit (**D**) or W53 (**E**) side chain. The isoquinoline moiety establishes -stacking with W53 and W96.

TRACER	BINDING POSE
LEGEND	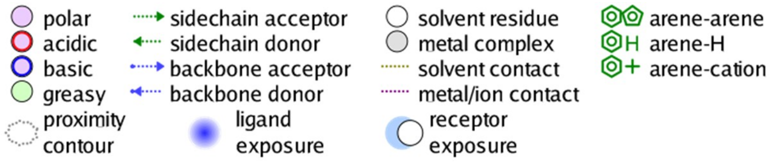
1. AC-5216 (Similar scaffold as DAC)	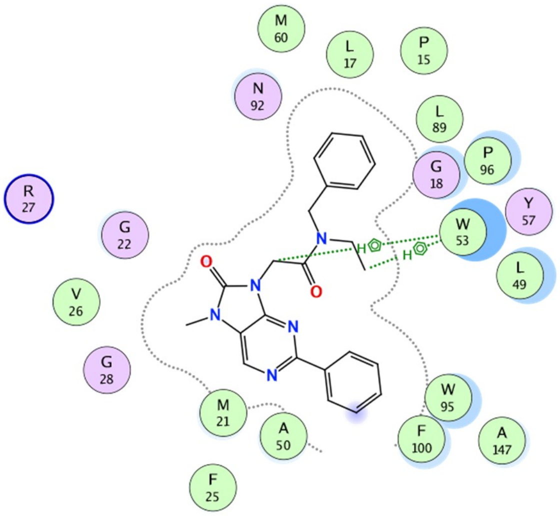
2. CB148	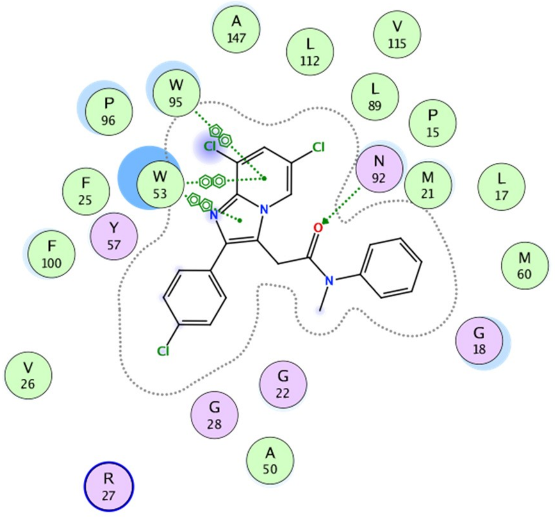

3. CLINME	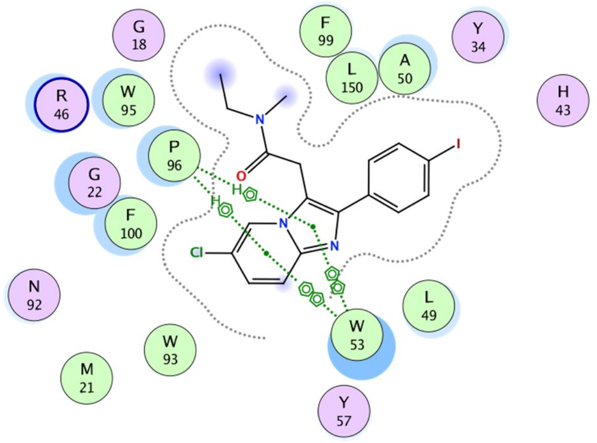
4. DPA-714 (Similar scaffold as DPA-713)	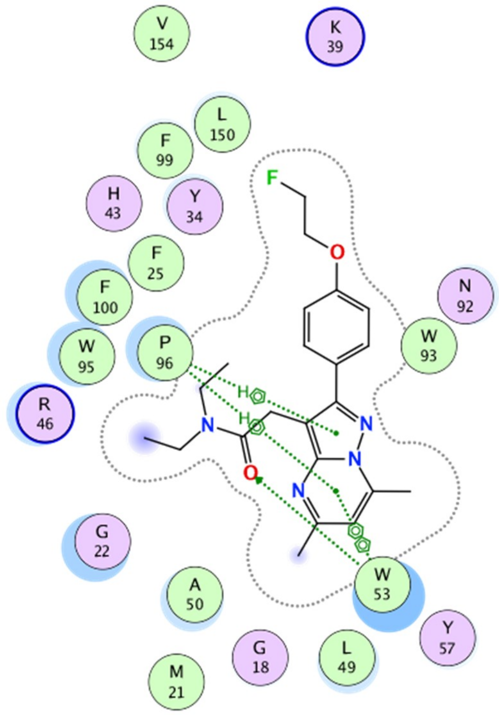

5. FEDAA1106 (Similar scaffold as DAA1106)	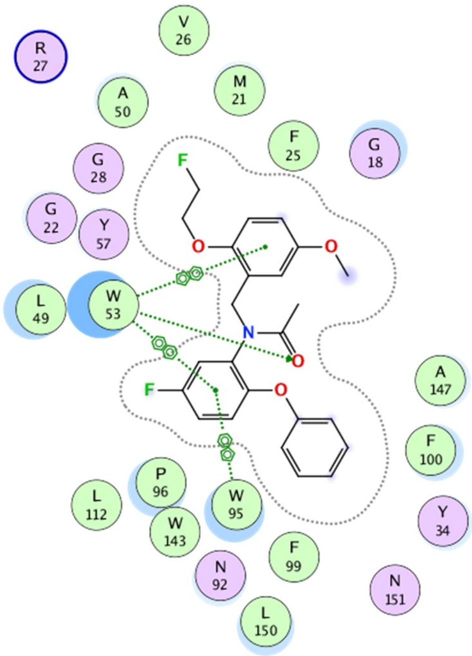
6. FGE-180	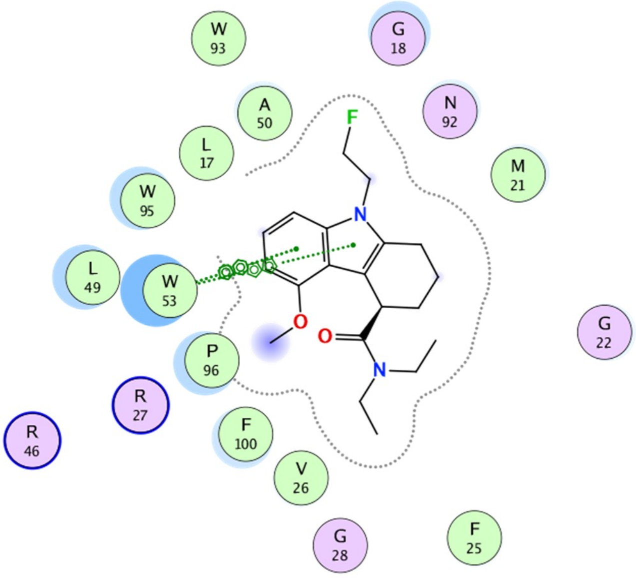

7. PBR111(Similar scaffold as PBR06 and PBR28)	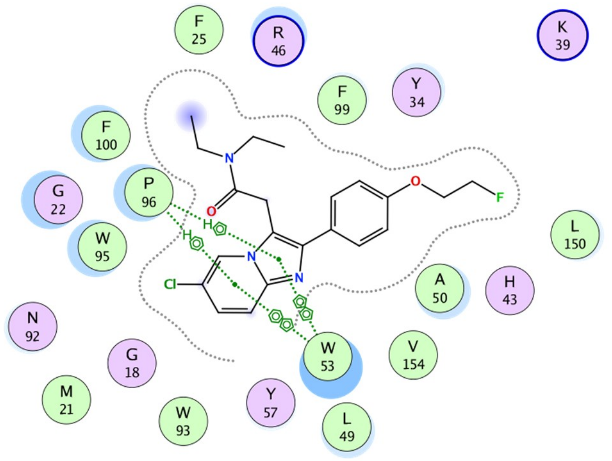
8. Protoporphyrin IX	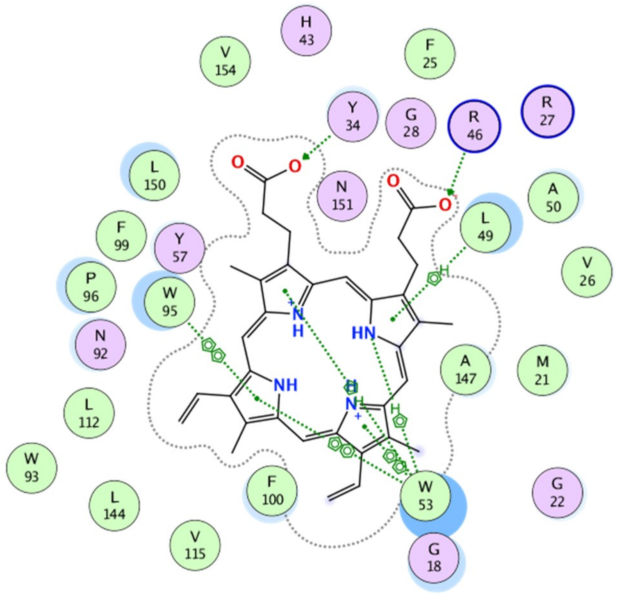
9. Ro5-486	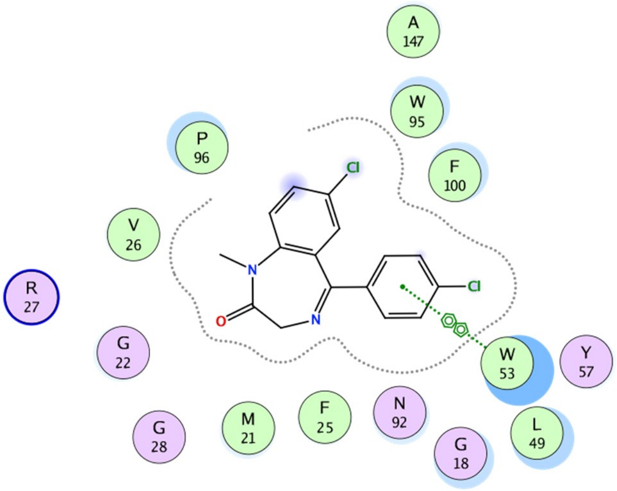

10. Vinpocetine	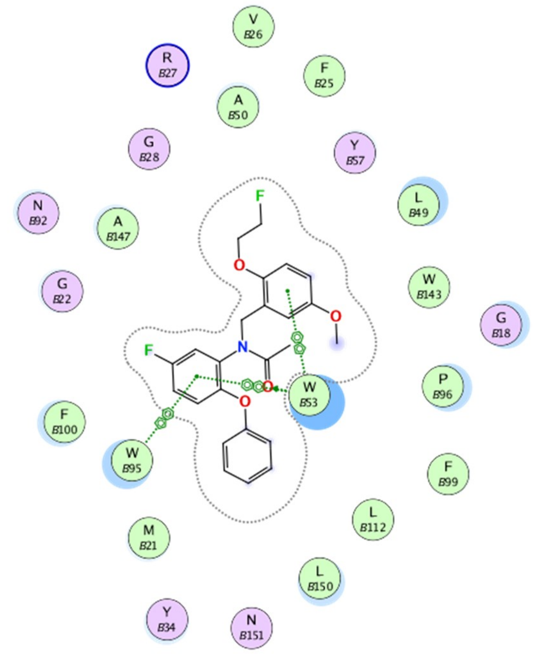

Table S7. Predicted binding poses of ligands known to bind to mammalian TSPO with nM affinity [12]: these are AC-5216, CB148, CLINME, DPA-714, FEDAA1106, FGE-180, PBR111, protoporphyrin IX, Ro5-486, and vinpocetine. The ligands have been docked in the structure of mTSPO_Rs using the Induced-fit docking algorithm implemented in the MOE code [13,14]. Overall, we notice that the main protein-ligand interactions involve W53, W95, and P96, through -stacking or H-bond interactions as in PK11195. In contrast to PK11195, the main difference that can be noticed is that the residue W95, belonging to the W95XXP97XF99 motif, directly establishes -stacking interactions with several ligands (namely, CB148, FEDAA1106, protoporphyrin IX, and vinpocetine). Please notice that water molecules are not considered here. During the MD simulations of TSPO_NMR monomer we did observe the participation of water molecules in the binding of the ligand to the binding pocket (Figure S11). Therefore, we cannot exclude that water molecules might also participate to the binding of those ligands in our model.

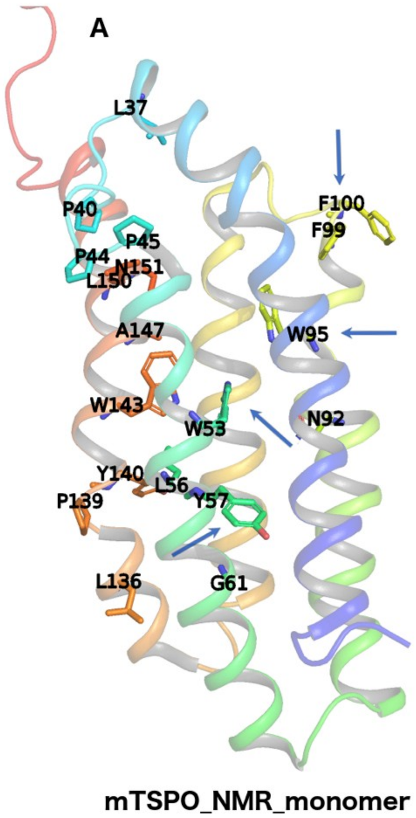

Figure S3. (**A**) mTSPO_NMR_monomer structure (PDBiD: 2MGY) [9]. (**B**) mTSPO_Rs_monomer structure. The side chains of highly-conserved residues are shown. The arrows indicate the residues with the largest variations in the side chain orientation between the two structures.

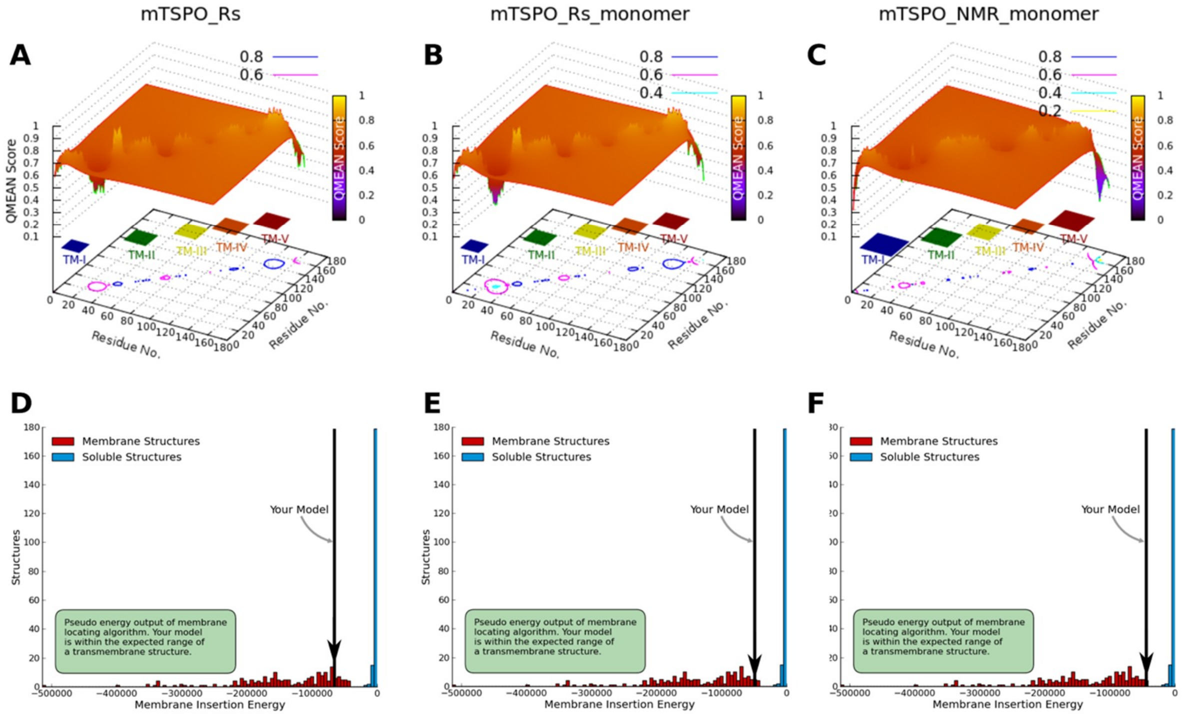

Figure S4. Evaluation of the membrane insertion of different mTSPO structures using the QMEANBrane [15] scoring function. QMEAN is a composite scoring function able to derive local (i.e., per residue) absolute quality estimates on the basis of one single model. QMEAN range from 1 (best score) to zero (the worst). QMEANBrane is

the version of QMEAN specialized for the evaluation of membrane proteins' structure quality. (**A–C**) show the QMEANBrane score plot for mTSPO_Rs, mTSPO_Rs_monomer, and mTSPO_NMR_monomer, respectively. The polygons represent the location of TM-I to TM-V helices and the contour lines represents the QMEAN score on XY plane diagonal. (**D–F**) show the membrane insertion energy plot for mTSPO_Rs, mTSPO_Rs_monomer, and mTSPO_NMR_monomer, respectively. The arrow represents the membrane insertion energy (lower values indicate better membrane embedding of the receptor). Plots (**D**) and (**E**) show better QMEANBrane scores than plot (**F**). The poor membrane embedding of the dimer mTSPO_NMR (Figure 2B in the main text) hampers any QMEANBrane-based calculation and, therefore, it is not possible to report the results for this structure in this picture.

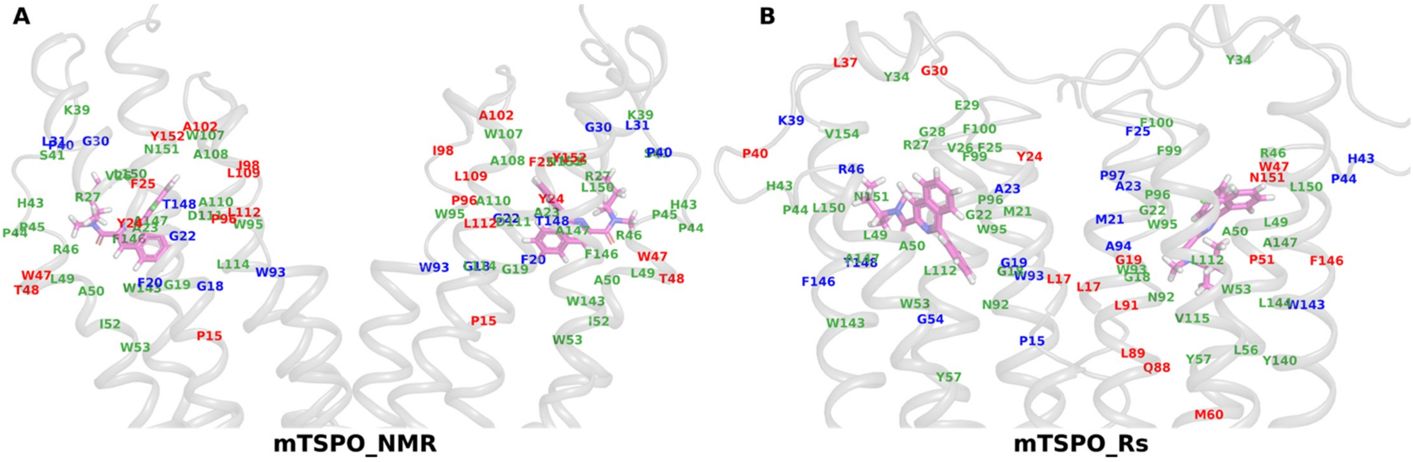

Figure S5. Residues within 4Å (green), 5Å (blue), and 6Å (red) from the PK11195 ligand (in pink) in mTSPO_NMR (**A**) and mTSPO_Rs **(B**).

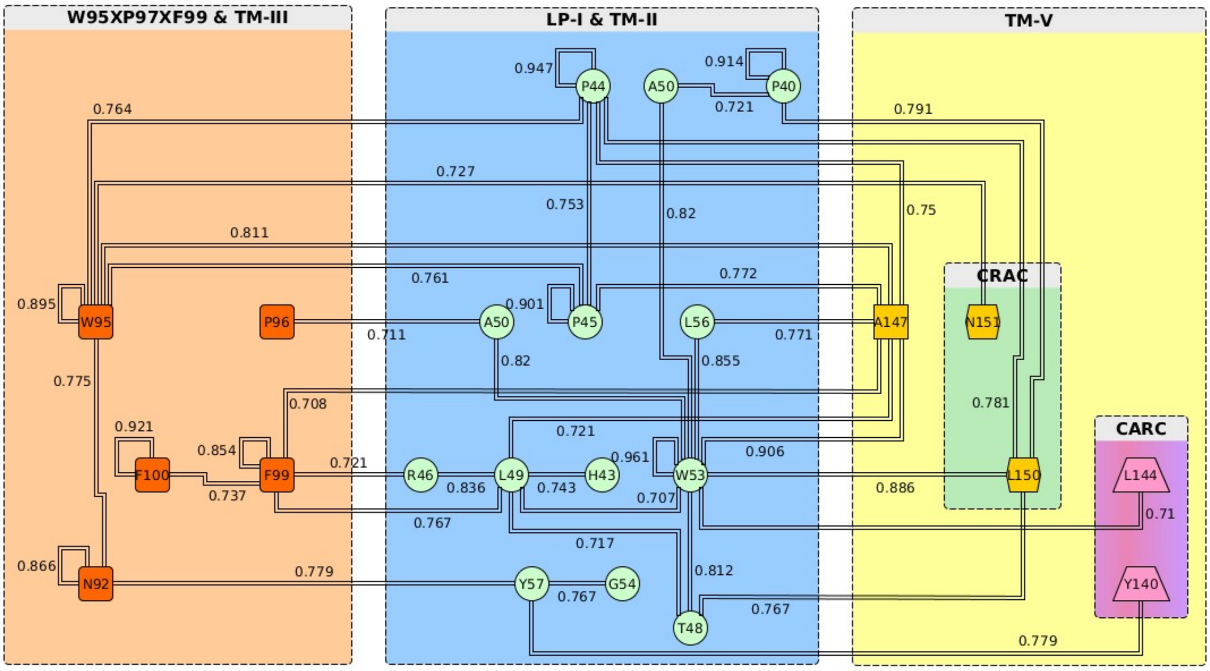

Figure S6. Residue pairs of coevolution and their pairwise χ2 scores, defined in [1]. The cutoff value on the χ2 score [1] is greater than 0.7 to identify the major components of pairwise coevolution. The analysis has been performed using the CoeViz code [3].

2. Molecular Dynamics Simulations

Seven-hundred nanosecond MD simulations were performed on mTSPO_NMR_monomer in the free state and in complex with the PK11195 ligand (in this section, apo and holo, respectively), starting from the deposited NMR structure [9]. The root-mean square deviation of the backbone atoms (N, Cα, C atoms, bb-RMSD) of mTSPO_NMR_monomer (excluded the highly flexible M1-W5 region located at N-tail and S159-E169 region located at the C-tail), LP-I (residues ranging from 37 to 45), LP-II (residue ranging from 72 to 75), LP-III (residues ranging from 100 to 103), LP-IV (residues ranging from 129 to 131), and PK11195 are plotted in Figure S7. The plots of these bb-RMSDs, as well as of the secondary structure, as a function of simulated time, suggest that the two systems equilibrate after about 400 ns (Figure S7 and Figure S8). All the analyses hereafter reported are, therefore, performed on the equilibrated part of our simulations.

Overall structural features. The average values for bb-RMSD of apo and holo mTSPO_NMR_monomer are 3.8 Å and 4.9 Å, respectively (Figure S7). This points to a higher flexibility of the holo structure with respect to the apo one. Notably, this flexibility is also reflected at the level of the secondary structure elements. The bb-RMSD of each helix is higher for holo mTSPO_NMR_monomer with respect to that in the apo structure. The secondary structure is mostly preserved for both apo and holo structures (Figure S8), with the exception of TM-II of the holo mTSPO_NMR_monomer, where a loss of about 10% of α-helix is observed with respect to the apo structure. The flexibility analyses were carried out by calculating the so-called PAD index [16]. The holo structure appears globally more flexible than the apo (Figure S9). The bending of each helix as a function of simulated time is presented in the main text (Figure 4).

Binding site analysis. The NMR binding pose of PK11195 is fairly preserved during the simulation (RMSD calculated over ligand and backbone atoms of the binding site is 4.9 Å relative to the starting configuration of the simulation). The residues in contact with PK11195 in the simulation are: F20, **A23**, L31, H43, P44, **R46**, **L49**, W53, N92, **W95**, **I98**, F99, W107, A109, **A110**, L113, and L114 (the residues in boldface have a contact percentage with the ligand higher than 90%, see Figure S10). The contacts with A23, L49, W107, A110, and L114, present in the deposited NMR ensemble [9], are retained throughout the simulation. On the contrary, the contacts between PK11195 and V26, A50, I52, A147, and L150, also present in the NMR ensemble [9], are lost during the simulation. The interactions between the ligand and the binding pocket are only hydrophobic (Figure S10 and Figure S11). This is consistent with the findings reported in ref. [9]. However, transient H-bonds are established within the water molecules with a total occurrence of about 74% (Figure S11).

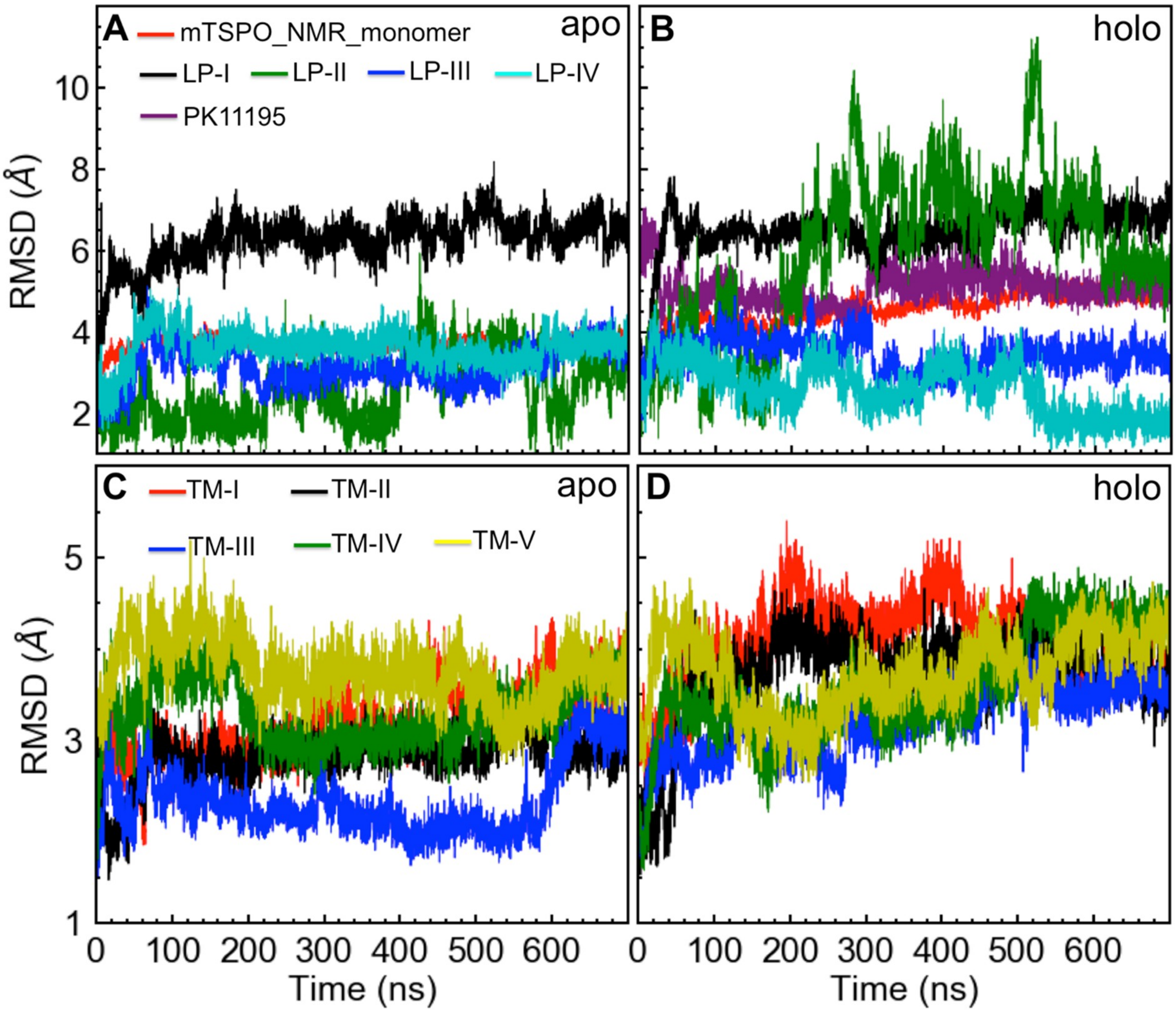

Figure S7 bb-RMSD of **apo** and all its loops (**A**)**,** of holo, all its loops, and PK11195 (**B**)**,** of each helix of apo (**C**) and of each helix of holo (**D**)**,** plotted as function of the simulation time. The bb-RMSD is computed with respect to the 13th frame from the NMR ensemble with PDBiD: 2MGY [9].

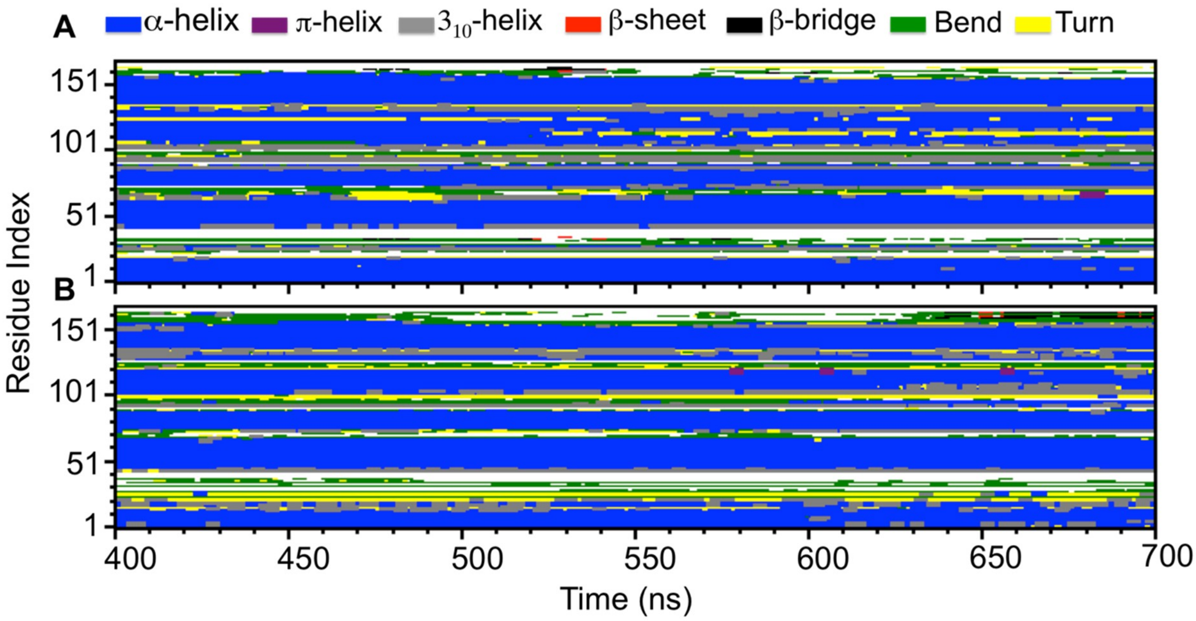

Figure S8. Secondary structures of apo (**A**) and holo (**B**) plotted as a function of the simulation time.

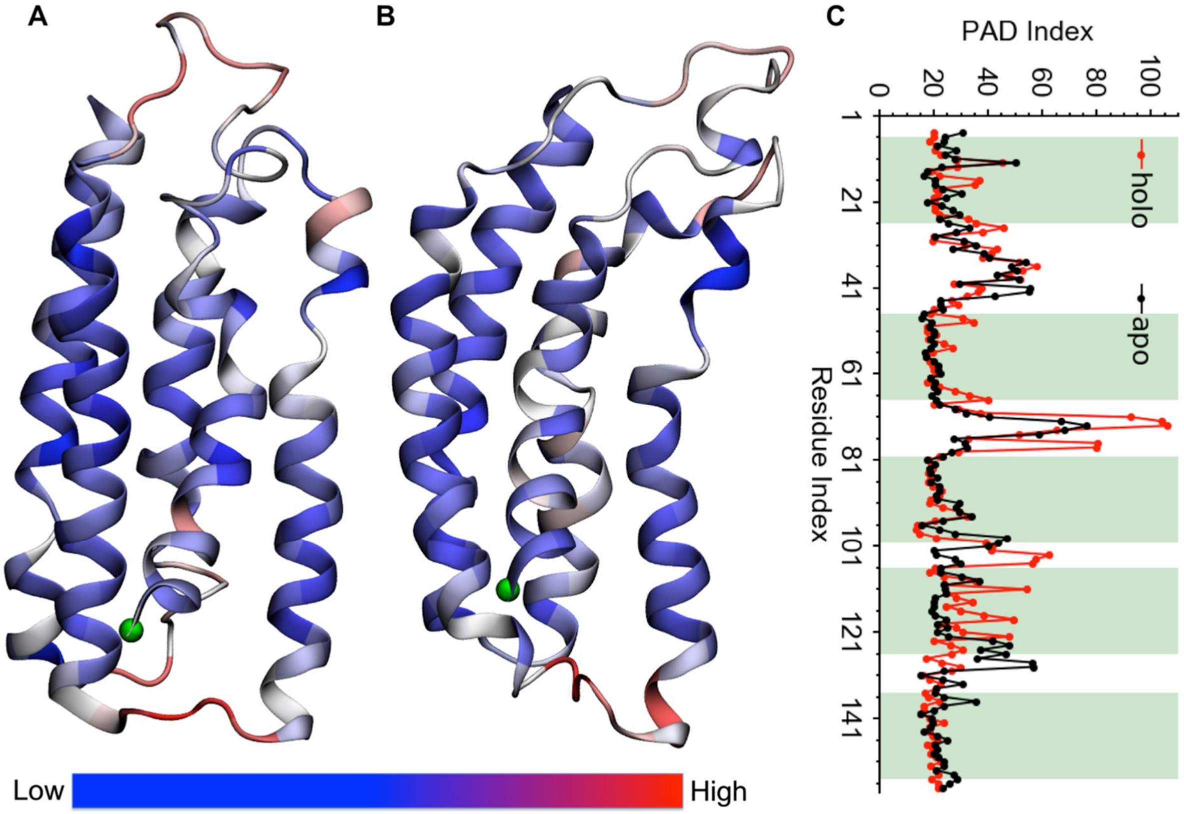

Figure S9. MD representative of apo (**A**) and holo (**B**) colored according to theirPAD. The N-terminus is indicated by a green sphere. (**C**) Plot of the PAD index of for each residue of the two systems. The helical regions present in the mTSPO structure taken from the NMR ensemble with PDBiD: 2MGY[9] are represented by the green shadow fields.

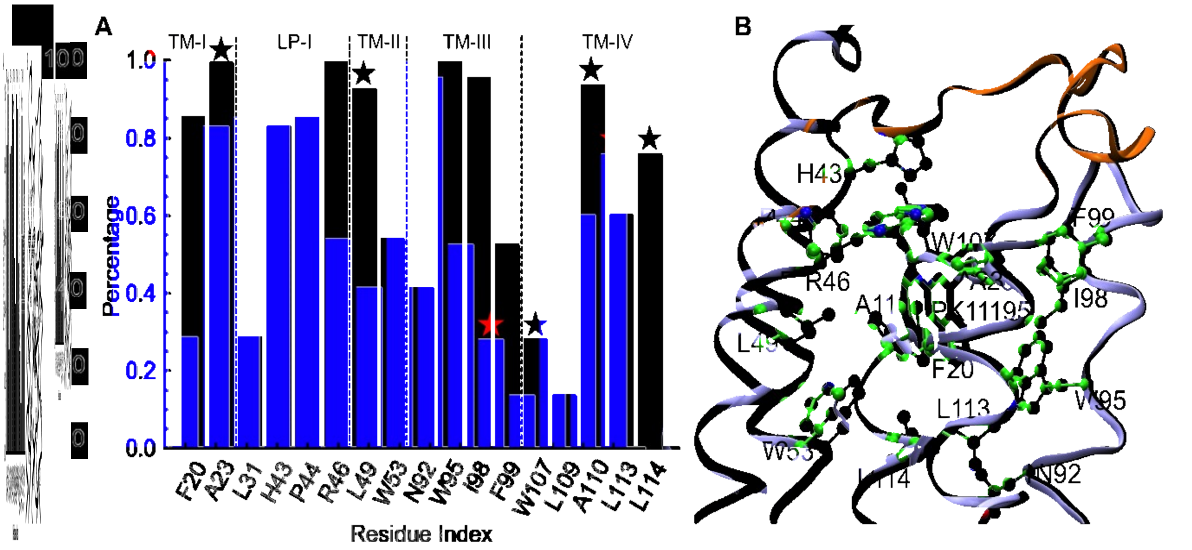

Figure S10. (**A**) Percentage of the contacts between PK11195 and holo mTSPO_NMR_monomer occurring during the simulation. Only residues with a percentage of contact larger than 10% of the entire simulation time are shown. The residues interacting with PK11195 in the deposited NMR ensemble with PDBiD: 2MGY [9] are marked by red stars. (**B**) The 3D structure of the binding pocket of mTSPO_NMR_monomer, as emerging from our MD simulation. The most populated cluster is shown. PK11195 and the protein residues are licorice and ball-stick, respectively. The LP-I loop is highlighted in orange cartoon, while the rest of the protein is in cyan.

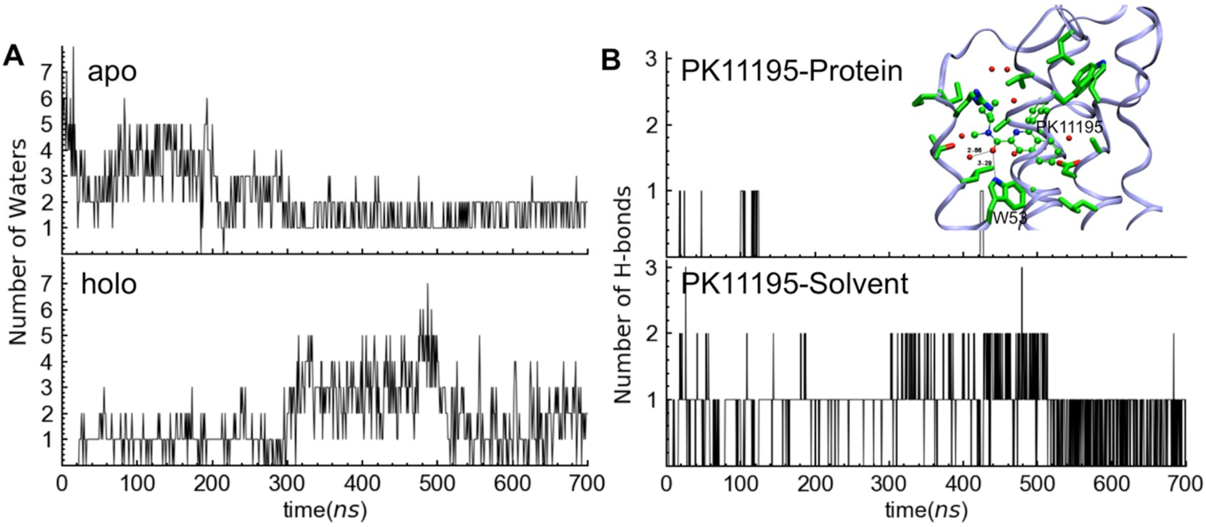

Figure S11. The number of water molecules (**A**) and of H-bonds (**B**) in the binding pocket of apo and holo is plotted as a function of the simulated time. In the inset figure, PK11195 and the protein residues in the MD representative structure are in ball-stick and licorice, respectively. Water molecules and H-bonds are represented by red spheres and dotted lines, respectively.

Supplementary References

1. Larson, S.M., Di Nardo, A.A., and Davidson, A.R., Analysis of covariation in an SH3 domain sequence alignment: applications in tertiary contact prediction and the design of compensating hydrophobic core substitutions. *J. Mol. Biol.*, **2000**, *303*, 433-446, doi: 10.1006/jmbi.2000.4146.
2. Sievers, F. and Higgins Desmond, G., Clustal Omega for making accurate alignments of many protein sequences. *Protein Sci.*, **2017**, *27*, 135-145, doi: 10.1002/pro.3290.
3. Baker, F.N. and Porollo, A., CoeViz: a web-based tool for coevolution analysis of protein residues. *BMC Bioinformatics*, **2016**, *17*, 119-119, doi: 10.1186/s12859-016-0975-z.

4. Bennet, S.A., Cohen, M.A., and Gonnet, G.H., Amino acid substitution during functionally constrained divergent evolution of protein sequences. *Protein Eng. Des. Sel.*, **1994**, *7,* 1323-1332, doi: 10.1093/protein/7.11.1323.
5. Lomize, M.A., et al., OPM database and PPM web server: resources for positioning of proteins in membranes. *Nucleic Acids Res.*, **2012**, *40,* D370-D376, doi: 10.1093/nar/gkr703.
6. Lomize Andrei, L., et al., Positioning of proteins in membranes: A computational approach. *Protein Sci.*, **2009**, *15,* 1318-1333, doi: 10.1110/ps.062126106.
7. Zimmermann, L., et al., A Completely Reimplemented MPI Bioinformatics Toolkit with a New HHpred Server at its Core. *J. Mol. Biol.*, **2017**, doi: 10.1016/j.jmb.2017.12.007.
8. Jaipuria, G., et al., Cholesterol-mediated allosteric regulation of the mitochondrial translocator protein structure. *Nat. Commun.*, **2017**, *8,* 14893, doi: 10.1038/ncomms14893.
9. Jaremko, L., et al., Structure of the Mitochondrial Translocator Protein in Complex with a Diagnostic Ligand. *Science*, **2014**, *343,* 1363-1366, doi: 10.1126/science.1248725.
10. Li, F., et al., Crystal Structures of Translocator Protein (TSPO) and Mutant Mimic of a Human Polymorphism. *Science*, **2015**, *347,* 555-558, doi: 10.1126/science.1260590.
11. Guo, Y., et al., Structure and Activity of Tryptophan-rich TSPO Proteins. *Science*, **2015**, *347,* 551-555, doi: 10.1126/science.aaa1534.
12. Trapani, A., et al., Targeting of the Translocator Protein 18 kDa (TSPO): A Valuable Approach for Nuclear and Optical Imaging of Activated Microglia. *Bioconjugate Chem.*, **2013**, *24,* 1415-1428, doi: 10.1021/bc300666f.
13. Chemical Computing Group, U.L.C., *Molecular Operating Environment (MOE)*. 2017: Montreal, QC, Canada.
14. Corbeil, C.R., Williams, C.I., and Labute, P., Variability in docking success rates due to dataset preparation. *J. Comput. Aided Mol. Des.*, **2012**, *26,* 775-786, doi: 10.1007/s10822-012-9570-1.
15. Studer, G., Biasini, M., and Schwede, T., Assessing the local structural quality of transmembrane protein models using statistical potentials (QMEANBrane). *Bioinformatics*, **2014**, *30,* i505-i511, doi: 10.1093/bioinformatics/btu457.
16. Caliandro, R., Rossetti, G., and Carloni, P., Local Fluctuations and Conformational Transitions in Proteins. *J. Chem. Theory Comput.*, **2012**, *8,* 4775-85, doi: 10.1021/ct300610y.
